# Extracellular vesicles‐encapsulated let‐7i shed from bone mesenchymal stem cells suppress lung cancer *via* KDM3A/DCLK1/FXYD3 axis

**DOI:** 10.1111/jcmm.15866

**Published:** 2020-12-22

**Authors:** Jiefeng Liu, Yuhua Feng, Xinyu Zeng, Miao He, Yujing Gong, Yiping Liu

**Affiliations:** ^1^ Department of General Surgery The Fourth Hospital of Changsha, Hunan Normal University Changsha China; ^2^ Department of Oncology the Second Xiangya Hospital, Central South University Changsha China; ^3^ Department of Oncology Xiangya Hospital, Central South University Changsha China

**Keywords:** bone marrow mesenchymal stem cell, doublecortin‐like kinase 1, extracellular vesicles, FXYD domain‐containing ion transport regulator 3, let‐7i, lung cancer, lysine demethylase 3A

## Abstract

Accumulating evidence has suggested that extracellular vesicles (EVs) play a crucial role in lung cancer treatment. Thus, we aimed to investigate the modulatory role of bone marrow mesenchymal stem cell (BMSC)‐EV‐derived let‐7i and their molecular mechanism in lung cancer progression. Microarray‐based analysis was applied to predict lung cancer‐related miRNAs and their downstream genes. RT‐qPCR and Western blot analyses were conducted to determine Let‐7i, lysine demethylase 3A (KDM3A), doublecortin‐like kinase 1 (DCLK1) and FXYD domain‐containing ion transport regulator 3 (FXYD3) expressions, after which dual‐luciferase reporter gene assay and ChIP assay were used to identify the relationship among them. After loss‐ and gain‐of‐function assays, the effects of let‐7i, KDM3A, DCLK1 and FXYD3 on the biological characteristics of lung cancer cells were assessed. Finally, tumour growth in nude mice was assessed by xenograft tumours in nude mice. Bioinformatics analysis screened out the let‐7i and its downstream gene, that is KDM3A. The findings showed the presence of a high expression of KDM3A and DCLK1 and reduced expression of let‐7i and FXYD3 in lung cancer. KDM3A elevated DCLK1 by removing the methylation of H3K9me2. Moreover, DCLK1 suppressed the FXYD3 expression. BMSC‐EV‐derived let‐7i resulted in the down‐regulation of KDM3A expression and reversed its promoting role in lung cancer development. Consistently, in vivo experiments in nude mice also confirmed that tumour growth was suppressed by the BMSC‐EV‐derived let‐7i. In conclusion, our findings demonstrated that the BMSC‐EV‐derived let‐7i possesses an inhibitory role in lung cancer progression through the KDM3A/DCLK1/FXYD3 axis, suggesting a new molecular target for lung cancer treatment.

## 1 INTRODUCTION

Lung cancer is the most prevalent cancer and the leading cause of cancer‐related death, accounting for over 2 million cases and approximately 1.76 million deaths in 2018 globally and has been a significant burden on health care around the world.^1^, ^2^ Risk factors for lung cancer include tobacco consumption, air contamination, second‐hand exposure to tobacco, genetic mutations and single nucleotide polymorphisms.^3^ Current treatments for lung cancer include surgery, radiation, chemotherapy and molecular‐targeted therapy; however, further understanding of the immune landscape of malignancies is required to enhance the therapeutic effects of molecular‐targeted therapy.^4^ Hence, this study was designed to explore the therapeutic target at the molecular level to alleviate lung cancer.

Extracellular vesicles (EVs) are tiny, subcellular sacs released in both physiological and pathological conditions, their function including carrying biological cargos derived from parent cells.^5^, ^6^ EVs derived from mesenchymal stem/stromal cells (MSCs) were identified to be involved in numerous lung pathologies, such as acute lung injury, acute respiratory distress syndrome and lung carcinoma.^7^, ^8^, ^9^ Most importantly, EVs derived from bone marrow mesenchymal stem cells (BMSCs) have been implicated in the development of lung cancer.^10^ The Let‐7 family is a group of microRNAs (miRNAs) that have been confirmed as key regulators of several physiological processes and immune responses of several cancers.^11^, ^12^, ^13^ The Let‐7i is a vital member of the let‐7 family and characterized as the premier miRNAs found to have an aberrant expression in multiple malignant tumours.^14^ For instance, according to a previous study, sera obtained from smokers and lung cancer patients was observed to have significantly down‐regulated levels of let‐7i‐3p, which is indicative of its association with the pathogenesis of smoking and smoking‐related lung cancer.^15^ Moreover, the miRNAs from EVs have been proposed as potential biomarkers for several diseases.^16^ Accordingly, let‐7a in serum exosomes has been reported to be involved in the epithelial‐to‐mesenchymal transition process and could be implicated in the treatment of metastatic melanoma.^17^ Notably, let‐7i‐5p within EVs has been illustrated as a tumour suppressor of head and neck squamous cell carcinoma.^18^ Nevertheless, the histone lysine demethylase 3A (KDM3A) is a histone demethylase in the JmjC domain‐containing protein family and has been associated with the development of tumours due to its ability to enhance gene transcription by demethylating H3K9me1 and H3K9Me2.^19^, ^20^, ^21^ Moreover, KDM3A has been indicated as a crucial factor in lung adenocarcinoma.^22^ On the other hand, doublecortin‐like kinase 1 (DCLK1), a cancer stem cell marker, is accounted for pathogenesis, development and poor prognosis in numerous types of cancer including non‐small cell lung cancer.^23^, ^24^ The specificity of DCLK1‐42 mAb and DCLK1‐87 mAb in NCM460, HCT116 and colorectal cancer tissues has been previously confirmed, and a high degree of overlap was observed between DCLK1 and microtubule protein expression, indicating that both DCLK1‐42 mAb and DCLK1‐87 mAb recognized DCLK1 in the cytoplasm.^25^ Of note, the clinical significance of FXYD domain‐containing ion transport regulator 3 (FXYD3), a sodium‐potassium ATPase regulator, has been demonstrated in several types of cancer.^26^ Particularly, the FXYD3 has been confirmed as a promising regulator in the progression of lung cancer.^27^ With the aforementioned findings taken into consideration, we conducted the present study with the aims of elucidating the regulatory role of EV‐let‐7i in lung cancer with the involvement of KDM3A/DCLK1/FXYD3 for finding a novel target for lung cancer treatment.

## 2 MATERIAL AND METHODS

### 2.1 Ethics statement

The current study was performed in accordance with the Declaration of Helsinki. Written informed consent was obtained from each participant. All animal experiments were performed with the approval of the Animal Committee of Xiangya Hospital Central South University and the recommendations of the Guide for the Care and Use of Laboratory Animals of the National Institutes of Health.

### 2.2 Study subjects

Cancer tissues and their corresponding paracancerous tissues (more than 5 cm from the cancer tissues) were obtained from 65 lung cancer patients (37 males and 28 females, aged 29‐78 years old) in Xiangya Hospital Central South University from March 2016 to March 2019. According to the classification criteria of the World Health Organization in 2015, there were 6 cases of adenocarcinoma and 14 cases of squamous cell carcinoma, with other types of lung cancer accounting for the remaining cases. The inclusion criteria for the patients were as follows: (a) patients without other malignant tumours; (b) patients received no chemotherapy, radiotherapy or other treatment before taking part in this study; (c) all collected samples were checked and confirmed by the pathologist. The matching criteria for the control group were as follows: (a) patients diagnosed as chronic pneumonia in Xiangya Hospital Central South University at the same time as the cancer cases; (b) patients who were willing to participate in a case‐control study.

### 2.3 Immunohistochemistry (IHC)

Lung cancer tissues and paracancerous tissues were embedded by paraffin and sectioned into 4‐μm‐thick sections. Paraffin‐embedded tissue sections were deparaffinized and hydrated. Citric acid (pH 6) was used to extract antigens for 30 minutes at 97°C after which the antigens were treated with 3% H_2_O_2_. Then, the tissue sections were incubated with the anti‐rabbit antibodies against FXYD3 (ab205534, 1:400, Abcam), KDM3A (ab191387, 1:500, Abcam) and DCLK1 (ab109029, 1:500, Abcam) overnight at 4℃. Afterwards, the sections were incubated with the horseradish peroxidase (HRP)‐conjugated secondary antibody (Dako; Agilent Technologies, Inc) for 30 minutes. Following 10‐minute incubation with 3,3'‐diaminobenzidine tetrahydrochloride, the sections were re‐stained with haematoxylin for 2 minutes. Finally, the pathology results were obtained under a microscope.

### 2.4 BMSC treatment

Human BMSCs were purchased from American type culture collection (ATCC, Manassas, VA, USA) and cultured in Mesenchymal Stem Cells medium (HUXMA‐03011‐440, Cyagen Biosciences Inc) in an environment containing 5% CO_2_ with 90% humidity. Oligonucleotides with suppressed or overexpressed let‐7i were cloned into the lentiviral vector pLenti‐U6‐pgkpuro for BMSC infection for the purpose of suppressing or overexpressing let‐7i.

### 2.5 Isolation and identification of EVs

After 48 ‐ 72 hours of cell incubation, the culture medium was collected and the EVs were isolated by ultracentrifugation. An equal volume of plasma (1 mL) and filtered phosphate‐buffered saline (PBS) was mixed to reduce the viscosity of the solution before centrifugation. Briefly, the cell culture medium was centrifuged at 300 g for 10 minutes, 2000 g for 15 minutes, and 12 000 g for 30 minutes to aid the removal of the floating cells and cell debris followed by filtration through a 0.22 μm filter. The supernatant was further ultracentrifuged at 1 × 10^6^ g at 4℃ for 2 hours, washed in PBS, and subjected to the second ultracentrifugation under the same conditions. Finally, the pellet was resuspended in about 100 mL of PBS and stored at −80℃ for future use or immediate use.

### 2.6 Transmission electron microscope (TEM) observation

EV pellet obtained by ultracentrifugation was fixed in 2% glutaraldehyde overnight at 4℃, washed with PBS, fixed with 1% OsO4 for 1 h, dehydrated in ethanol and embedded using epoxy resin. The embedded material was sectioned using a microtome and saturated with sodium periodate and 0.1N hydrochloric acid. After 10 minutes, the size and morphological characteristics of EV were examined with the use of a TEM (JEM‐1010, JEOL, Tokyo, Japan). The EV suspension was mixed with an equal volume of 4% paraformaldehyde and deposited on a Formvar carbon‐coated EM grid. Images were acquired using a TEM (Hitachi, Tokyo, Japan). Western blot analysis was applied to identify the EV surface marker proteins of rabbit anti‐CD63 (ab134045, 1:1000, Abcam, Cambridge, UK), rabbit anti‐CD81 (ab109201, 1:5000, Abcam), and rabbit anti‐Calnexin (ab92573, 1:20 000, Abcam).

### 2.7 Nanoparticle tracking analysis (NTA)

The size distribution and concentration of EVs were determined by the NTA (Zetasizer Nano ZS90 instrument, Malvern Panalytical) according to the characteristics of light scattering and Brownian motion. The EVs were resuspended and mixed in 1 ml of PBS and the diluted EVs were injected into a Zetasizer Nano ZS90 instrument followed by the determination of particle size according to Brownian motion and diffusion coefficients. Filtered PBS was used as a control. All samples were measured with NP100 membrane using 44.5 mm and 0.64 V. Samples were diluted (1:1000) using CPC100 standard particles at the same settings. Collectively, five videos were recorded, usually lasting for 60 seconds. Subsequently, the data were analysed with Zetasizer software (Malvern Panalytical) and optimized to identify and track each particle frame by frame.

### 2.8 EV labelling and immunofluorescence

EVs were resuspended in 400 μL of PBS at a concentration of 0.1‐0.2 μg and stained with CellMask Deep Red (Thermo Fisher Scientific) with the excitation/emission wavelength of 649/666 nm. At the time of labelling, EVs were incubated with crimson dye (1:1000) at 37℃ for 20 minutes. Unbound dye was removed by PBS washing (1 to 10 000 v/v ratio), after which EVs were centrifuged at 100 000 × g for 1 hour and diluted in PBS. The protein concentration was determined using a BCA protein detection kit.

Cells were stained with the CellTrace^TM^ Carboxyfluorescein succinimidyl ester (CFSE, Life Technologies) with a maximum excitation/emission wavelength of 492/517 nm. The CFSE dyes diffuse into cells after being digested by the endoesterase and covalently bound to the intracellular amines to form stable fluorescent staining. About 3 to 5 × 10^5^, A549 cells in serum‐free medium were stained with CFSE dye at a ratio of 1:1000 (5 μM working concentration) and then incubated at 37℃ for 20 minutes avoiding light exposure. The solution was settled, and the cells were washed with a serum‐free medium at a ratio of 1:10 to remove the free dye.

Cells were inserted into an 8‐well chamber slide (Millipore). CFSE‐stained cells were incubated and treated with EVs at different time‐points. Cells were washed and fixed with 3.7% (w/v) formaldehyde for 5 minutes at room temperature for cell imaging. The observation was conducted using a fluorescence microscope.

### 2.9 Cell culture and transfection

Normal lung fibroblasts LL29 as well as lung cancer cell lines A549 and H125 were purchased from ATCC. LL29 and H125 cells were cultured in RPMI‐1640 (Gibco) containing 10% foetal bovine serum (FBS, Gibco), 10 μg/mL streptomycin and 100 U/mL penicillin in a 37°C, 5% CO_2_ incubator (Thermo Fisher Scientific), whilst A459 was cultured in Ham's F‐12K (Kaighn's) Medium. Cells in the logarithmic phase were trypsinized and inoculated in a 6‐well plate at a density of 1 × 10^5^ cells per well. After 24 hours of routine culture, the cells were transfected when the cell confluency reached approximately 75%, according to the instructions of the Lipofectamine 2000 (Invitrogen). Lung cancer cells were transfected with the BMSC‐EV, EV‐let‐7i‐inhibitor, EV‐let‐7i‐mimic, overexpressed (oe)‐KDM3A, oe‐FXYD3, oe‐DCLK1, silenced (si)‐KDM3A, si‐FXYD3, short hairpin RNA (sh)‐DCLK1‐1, sh‐DCLK1‐2 and their negative controls (EV‐inhibitor‐NC, EV‐mimic‐NC, oe‐NC, si‐NC, sh‐NC). The control cells were treated by PBS. After 48 hours of transfection, the transfection efficiency of each factor was detected by the reverse transcription‐quantitative polymerase chain reaction (RT‐qPCR). The plasmids used above were purchased from Shanghai GenePharma Biological Co., Ltd. (Shanghai, China). The plasmid concentration was 50 ng/mL.

### 2.10 RT‐qPCR

Total RNA was extracted using the TRIzol reagent (15596026, Invitrogen), RNA was reversely transcribed into cDNA according to the instructions of the PrimeScript^RT^ reagent Kit (RR047A, Takara), and the synthesized cDNA was determined by RT‐qPCR using Fast SYBR Green PCR reagent (Applied biosystems, Thermo Fisher Scientific) and ABI PRISM 7300 RT‐PCR system (Applied Biosystems). Each well was set up with 3 replicates. Glyceraldehyde phosphate dehydrogenase (GAPDH) was used as an internal reference, and the relative expression of MALAT1 was analysed using the 2^−ΔΔ^ *^C^* ^t^ method as follows: △△C*t *= (average *C*_t_ value of the target gene in the experimental group ‐ average *C*_t_ value of the housekeeping gene in the experimental group) − (average *C*_t_ value of the target gene in the control group − average *C*_t_ value of the housekeeping gene in the control group). The designed primers are illustrated in Table 1.

**Table 1:** Primer sequence of RT‐qPCR

Gene	Primer sequence
let‐7i	F: 5‐GGGGTGAGGTAGTAGTTTGT‐3
	R: 5‐TGCGTGTCGTGGAGTC‐3
KDM3A	F: 5‐TTCTTTTCCTCCAAGATTCCC‐3
	R: 5‐GGGACCATTCGAGCTGTTT‐3
DCLK1	F: 5‐TAGCCAGCGCCATCAAATAC‐3
	R: 5‐ACCCAGCTTCAGTGATTTGC‐3
FXYD3	F: 5‐CCTGTCCTGGACGCCAAT‐3
	R: 5‐GAGGCTGTGCCAGTCATAGTAGAA‐3
GAPDH	F: 5‐TGCACCACCAACTGCTTAGC‐3
	R: 5‐GGCATGGACTGTGGTCATGAG‐3
U6	5‐CACGAATTTGCGTGTCATCCTT‐3

Note

DCLK1, doublecortin‐like kinase 1; F, forward; FXYD3, FXYD domain‐containing ion transport regulator 3; GAPDH, glyceraldehyde phosphate dehydrogenase; KDM3A, lysine demethylase 3A; R, reverse; RT‐qPCR, reverse transcription‐quantitative polymerase chain reaction.

### 2.11 Western blot analysis

Cells were trypsinized and lysed with an enhanced radioimmunoprecipitation assay (RIPA) lysis buffer (Boster Biological Technology Co. Ltd.) containing a protease inhibitor, after which the protein concentration was determined using a bicinchoninic acid (BCA) protein quantification kit (Boster Biological Technology Co. Ltd.). Proteins were separated by 10% sodium dodecyl sulphate‐polyacrylamide gel electrophoresis (SDS‐PAGE). The separated proteins were electrotransferred to a polyvinylidene fluoride (PVDF) membrane (Millipore). The membrane was blocked with 5% bovine serum albumin (BSA) at room temperature for 2 hours to block non‐specific binding and incubated with diluted primary antibodies as follows: mouse anti‐KDM3A (ab91252), rabbit anti‐DCLK1 (ab37994), rabbit anti‐FXYD3 (ab205534), rabbit anti‐vimentin (ab92547), rabbit anti‐N‐cadherin (ab18203), rabbit anti‐E‐cadherin (ab76319), rabbit anti‐β‐actin (ab8227) and mouse anti‐β‐actin (ab8226) overnight at 4℃ and then cultured with HRP‐labelled goat anti‐rabbit secondary antibody (ab205718) or goat antimouse secondary antibody (ab205719) for 1 hour at room temperature. All antibodies used above were obtained from Abcam. Subsequently, the membrane was developed with ECL working solution (Millipore). The ImageJ analysis software (Bio‐Rad) was used to quantify the grey levels of each band in the Western blot analysis and β‐actin was used as an internal reference.

### 2.12 Cell counting kit 8 (CCK‐8) assay

The CCK‐8 kit (Dojindo) was used according to the manufacturer's instructions. A549 or H125 cells were seeded on a 96‐well plate with different EVs. Under cell culture conditions, each well was added with CCK‐8 solution at 0, 24, 48, 72 hours, respectively, followed by 2 hours of incubation. Viable cells were measured with a microplate reader (Multiskan Sky Microplate Spectrophotometer, Cat. No. 51119570, Thermo Scientific) at an optimal density of 450 nm.

### 2.13 Transwell assay

The apical chamber of the bottom membrane of the Transwell chamber was coated with Matrigel (BD Biosciences), and the Matrigel was polymerized into a gel at 37°C for 30 minutes. The base membrane was hydrated before use. The cells were cultured in serum‐free medium for 12 hours and then harvested and resuspended in serum‐free medium (1 × 10^5^/mL). The basolateral chamber was added with 10% FBS and the Transwell chamber was added with 100 μL of the cell suspension. After incubation at 37℃ for 24 hours, cells that did not invade the surface of the Matrigel membrane were gently removed with a cotton swab, whilst cells that invaded the membrane were fixed with 100% methanol and stained with 1% toluidine blue (Sigma‐Aldrich). Stained invading cells were counted by hand using an inverted light microscope (CarlZeiss). Five fields of view were randomly selected for counting.

### 2.14 Flow cytometry

According to the manufacturer's instructions, apoptosis of A549 cells was detected by Annexin‐V‐fluorescein isothiocyanate (Annexin‐V‐FITC)/propidium iodide (PI) flow cytometry kit (BD Biosciences). Cells were washed twice with iced PBS and resuspended in 200 μL of binding buffer at a concentration of 1 × 10^6^ cells/mL. Cells were incubated in 10 μL PI and 10 μL Annexin‐V‐FITC under dark conditions at 4℃ for 30 minutes. Finally, 300 μL of binding buffer was added and analysed with the use of flow cytometer (Cytomics FC 500, Beckman Coulter) within 1 hour.

### 2.15 Dual‐luciferase reporter gene assay

KDM3A 3’‐UTR sequences containing a mutant (MUT) or wild‐type (WT) let‐7i binding site was designed and constructed by GenScript (Nanjing, China) and cloned into the pGL‐3 luciferase report vector. HEK293T cells were cotransfected with pGL‐3‐WT‐KDM3A or pGL‐3‐MUT‐KDM3A 3’‐UTR reporter plasmid and let‐7i mimic or mimic‐NC using the Lipofectamine 3000 (Invitrogen) after 24 hours of culture in a 24‐well plate. The dual‐luciferase reporter gene assay system (Promega, Madison, WI, USA) was applied in the measurement of the Firefly and Renilla luciferase activities. The relative activity of cells is expressed as the ratio of Firefly luciferase activity/Renilla luciferase activity.

### 2.16 Chromatin immunoprecipitation (ChIP) assay

After reaching about 70%‐80% of cell confluence, the cells were added with 1% formaldehyde and fixed at room temperature for 10 minutes to fix and cross‐link the DNA and protein in cells. After cross‐linking, cells were randomly fractured by ultrasonic treatment, each ultrasound was 120 w, 2 seconds on, 5 seconds off, and then it was circulated 15 times to break it into fragments of appropriate size. Then cells were centrifuged at 6540 *g* at 4°C with the supernatant collected. Afterwards, positive control antibody RNA polymerization Enzyme II, negative control antibody Normal human IgG and KDM3A antibody (ab91252, Abcam), H3K9me2 antibody (ab1220, Abcam) were added, respectively, to immunoprecipitate DNA/protein complex. After immunoprecipitation, the DNA was washed, reverse‐crosslinked, and protein was removed by proteinase K treatment. Eluted DNA was purified using the Active Motif's ChIP DNA purification kit (Cat. No. 58 002, Millipore), and qPCR was conducted to verify the DCLK1 promoter expression.

### 2.17 Xenograft tumours in nude mice

Twenty‐four healthy Balb/c nude mice (Beijing Institute of Pharmacology, Chinese Academy of Medical Sciences, Beijing, China) aged 6‐8 weeks were housed in a specific pathogen‐free (SPF) animal laboratory in different cages with a humidity of 60%‐65%, temperature 22°C‐25°C and provided with free food and water under 12‐hour light and dark cycle. The experiment was started one week after adaptive feeding, and the health status of nude mice was observed before the experiment. A xenograft model was established with the subcutaneous injection of 1 × 10^6^ A549 cells into the abdomen of nude mice. After successful modelling, they were randomly and equally divided into 4 groups, and treated differently as follows: PBS (500 μL of PBS was injected *via* tail vein every 2 d), EV‐let‐7i‐mimic (EVs were extracted after transfection of let‐7i‐mimic into BMSC, suspended in sterile PBS and injected into mice from tail vein every 2 days at 500 μL, 25 μg/mL each time), si‐FXYD3 (si‐FXYD3 adenoviral vectors were injected into nude mice from tail vein every 2 days at 500 μL, 25 μg/mL each time) and si‐FXYD3 + EV‐let‐7i‐mimic (EVs were extracted after transfection of let‐7i‐mimic into BMSC and EVs and si‐FXYD3 adenoviral vectors) were mixed and injected from tail vein every 2 days. The tumour volume of each mouse was measured with a vernier caliper. After A549 cell transplantation, the tumours were dissected and weighed, after which the mice were killed on the 30th day following these procedures. Haematoxylin‐eosin staining was used to detect lung metastasis on paraffin sections.

### 2.18 Statistical analysis

The SPSS 21.0 statistical software (IBM Corp.) was used for statistical analysis. Data were expressed as the mean ± standard derivation. Data of cancer tissues and paracancerous tissues were compared using paired *t* test, and data between the other two groups were compared using an unpaired *t* test. Comparison among multiple groups was analysed by one‐way analysis of variance (ANOVA). Comparison among groups at different time‐points was analysed by the two‐way ANOVA. Tumour volume was analysed by the repeated‐measures ANOVA *P* < .05 was considered statistically significant.

## 3 RESULTS

### 3.1 BMSC‐EV‐derived let‐7i inhibits proliferative, migrative and invasive potentials of lung cancer cells

**Figure 1:**
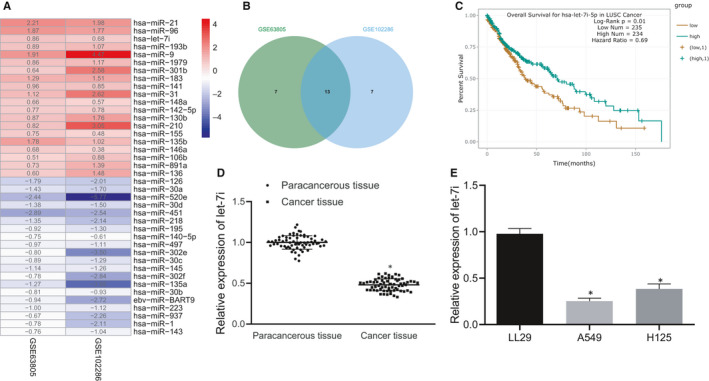
Let‐7i is lowly expressed in lung cancer tissues and cells. A, Differentially expressed miRNAs in microarray GSE63805 and GSE102286. Colour scale of log fold change (FC) value is on the right. B, The intersection of the top 20 miRNAs in microarray GSE63805 and GSE102286. C, The survival curve of let‐7i‐5p in three significantly related miRNA survival curves obtained from StarBase analysis of the relationship between intersection miRNAs and lung cancer prognosis. D, The let‐7i expression in lung cancer tissues determined by RT‐qPCR (n = 65), *P < .05 vs. paracancerous tissues. E, The expression of let‐7i in lung cancer cells A549 and H125, normal human lung fibroblasts LL29 determined by RT‐qPCR, *P < .05 compared with LL29 cell. Data are all measurement data and expressed as the mean ± standard deviation. The experiment was repeated 3 times

According to existing literature, mesenchymal stem cell (MSC)‐derived exosomes are capable of inhibiting lung cancer progression.^28^ To study its specific regulatory mechanism, miRNAs that were closely related to lung cancer were screened out. The difference analysis of lung cancer‐related miRNA expression microarray GSE63805 and GSE102286 obtained from Gene Expression Omnibus (GEO) database (https://www.ncbi.nlm.nih.gov/gds) was conducted through ‘limma’ package of R‐language (http://www.bioconductor.org/packages/release/bioc/html/limma.html). We observed that GSE63805 has 62 samples (Figure 1A) including 30 normal samples and 32 lung cancer samples, whereas GSE102286 had 179 samples including 88 normal samples and 91 lung cancer samples. The Venn diagram was plotted using the intersection among the top significantly differentially expressed 20 miRNAs in microarrays. The miRBase (http://www.mirbase.org) was used to obtain the miRNA suffix to get the full name of the intersection gene. The survival curves of each miRNA were obtained from the StarBase (http://starbase.sysu.edu.cn/) to determine which miRNAs are related to the prognosis of lung cancer and key miRNAs were selected based on existing literatures. A total of 20 miRNAs with the lowest *p* value were selected, and Venn diagram was plotted to take the intersection with 13 miRNAs obtained (Figure 1B). Databases RAID (Score > 0.6) (http://www.rna‐society.org/raid2/index.html), mirDIP (Integrated Score > 0.8) (http://ophid.utoronto.ca/mirDIP/), DIANA TOOLS (miTG score > 0.7) (http://diana.imis.athena‐innovation.gr/DianaTools), miRDB (Target Score > 85) (http://www.mirdb.org), starBase (clipExpNum > 10, pancancerNum > 5) and miRWalk (energy < −20, accessibility < 0.01, au > 0.1) (http://mirwalk.umm.uni‐heidelberg.de) were employed to predict the downstream genes of let‐7i and Venn diagram was plotted to get key genes. The key transcription factors were screened out by comparing key genes with human transcription factors obtained from Cistrome (http://cistrome.org). The binding site between miRNAs and genes was identified from the StarBase database. Based on the existing literature, the possible downstream pathways of key transcription factors were predicted, correlation analysis was performed by starBase, and co‐expression analysis by MEM (https://biit.cs.ut.ee/mem/index.cgi) was used to analyse downstream pathways. Then, the survival curve illustrated that let‐7i‐5p was closely related to the prognosis of lung cancer (Figure 1C, Table 2). Additionally, results from RT‐qPCR showed an evident decrease in the expression of let‐7i in lung cancer tissues when compared with that of paracancerous tissues (*P* < .05) (Figure 1D). Subsequently, the expression of let‐7i in lung cancer cells was determined and we observed that compared with that of human normal lung fibroblasts LL29, let‐7i expression was considerably lowered in lung cancer cells A549 and H125, whereas the expression was the lowest in A549 cells (Figure 1E). Therefore, lung cancer cell line A549 was selected for subsequent experiments.

**Table 2:** Survival analysis of each gene

miRNA	HR	*p* Value
miR‐451a	0.69	0.01
let‐7i‐5p	0.69	0.01
miR‐218‐5p	0.74	0.033
miR‐96‐5p	0.81	0.14
miR‐126‐3p	1.2	0.2
miR‐140‐5p	0.83	0.2
miR‐9‐5p	1.15	0.33
miR‐30d‐5p	0.89	0.44
miR‐520e‐3p	0.82	0.49
miR‐21‐5p	1.1	0.53
miR‐195‐5p	0.92	0.57
miR‐30a‐5p	0.95	0.74
miR‐193b‐3p	1.01	0.94

**Figure 2:**
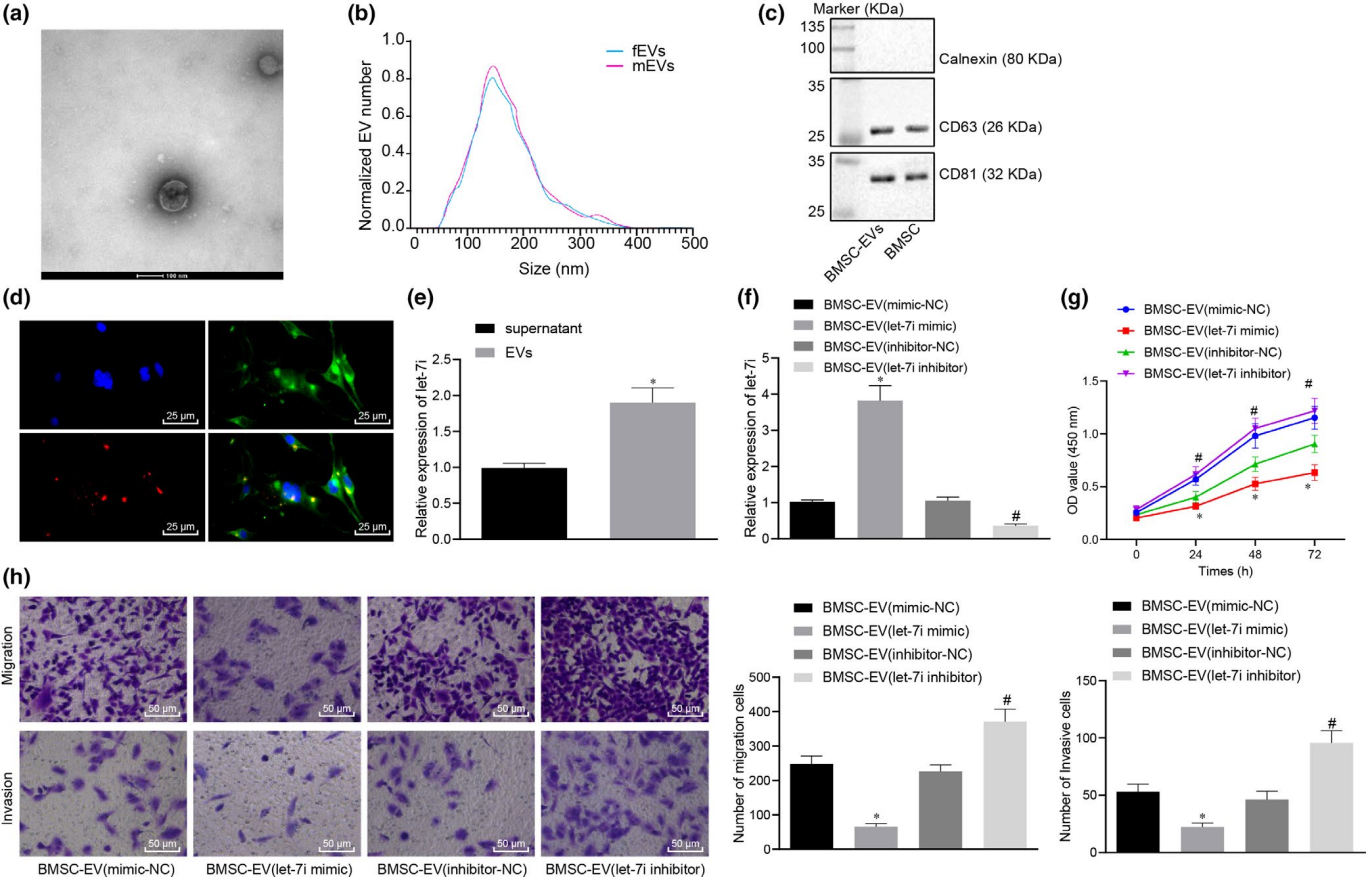
BMSC‐EV‐let‐7i‐mimic regulates lung cancer cell proliferation, migration and invasion negatively. A, BMSC‐EVs observed under TEM (Scale bar = 100 nm). B, The size distribution of EVs determined by NTA. C, BMSC‐EVs (5 μg) and BMSC lysate (20 μg) determined by Western blot analysis. D, Immunofluorescence overlay image of human lung cancer cells uptake EVs at 24th h (double label; red‐EVs‐20%; green‐lung cancer cells; × 400). E, Let‐7i expression in BMSC‐EVs and lysate supernatants measured by RT‐qPCR, *P < .05 compared with the supernatant. F, The expression of let‐7i in lung cancer cells treated with EVs determined by RT‐qPCR. G, The proliferation of lung cancer cells treated with EVs determined by CCK8. H‐I, The migration and invasion of lung cancer cells treated with EVs determined by Transwell (× 200), *P < .05 compared to cells treated by BMSC‐EV‐mimic‐NC; #*P* < .05 compared to cells treated by BMSC‐EV‐inhibitor‐NC. The experiment was repeated 3 times

Furthermore, the BMSC‐EVs were extracted and then identified with the application of TEM, NTA and Western blot analysis. It was revealed that BMSC‐EVs had an average diameter of 155 ± 2.8 nm and expressed CD63 and CD81 proteins but that did not express nanoparticles of calnexin protein (Figure 2A‐C). Next, lung cancer cells A549 were stained by CFSE. Whilst the BMSC‐EVs were stained with a deep red dye of lipophilic cell membrane. Then, 10 μg of the stained BMSC‐EVs were added to the CFSE‐stained lung cancer cells and cultured for a period of time. Our data from the fluorescence microscope revealed that lung cancer cells could adhere to and internalize/uptake EVs (Figure 2D).

The expression of let‐7i in BMSC‐derived EVs was determined, and we found that compared to the supernatant, let‐7i expression was remarkably higher in EVs (Figure 2E). BMSC‐EVs extracted from BMSCs were transfected with let‐7i mimic/let‐7i inhibitor and added into lung cancer cells in order to prove that let‐7i in EVs are capable of regulating the pathogenesis and development of lung cancer. The expression of let‐7i was measured by the RT‐qPCR. Our results demonstrated that the expression of let‐7i in lung cancer cells was remarkably increased in cells after treatment of BMSC‐EV‐let‐7i‐mimic, whereas the proliferative, migrative and invasive abilities of lung cancer cells were notably inhibited. However, the co‐culture of lung cancer cells with the BMSC‐EV‐let‐7i inhibitor led to opposite trends (Figure 2F‐I). In short, Let‐7i in BMSC‐EVs exerted an inhibitory effect on proliferative, migrative and invasive abilities of lung cancer cells.

### 3.2 BMSC‐EV‐derived let‐7i inhibits the pathogenesis of lung cancer by repressing KDM3A

**Figure 3:**
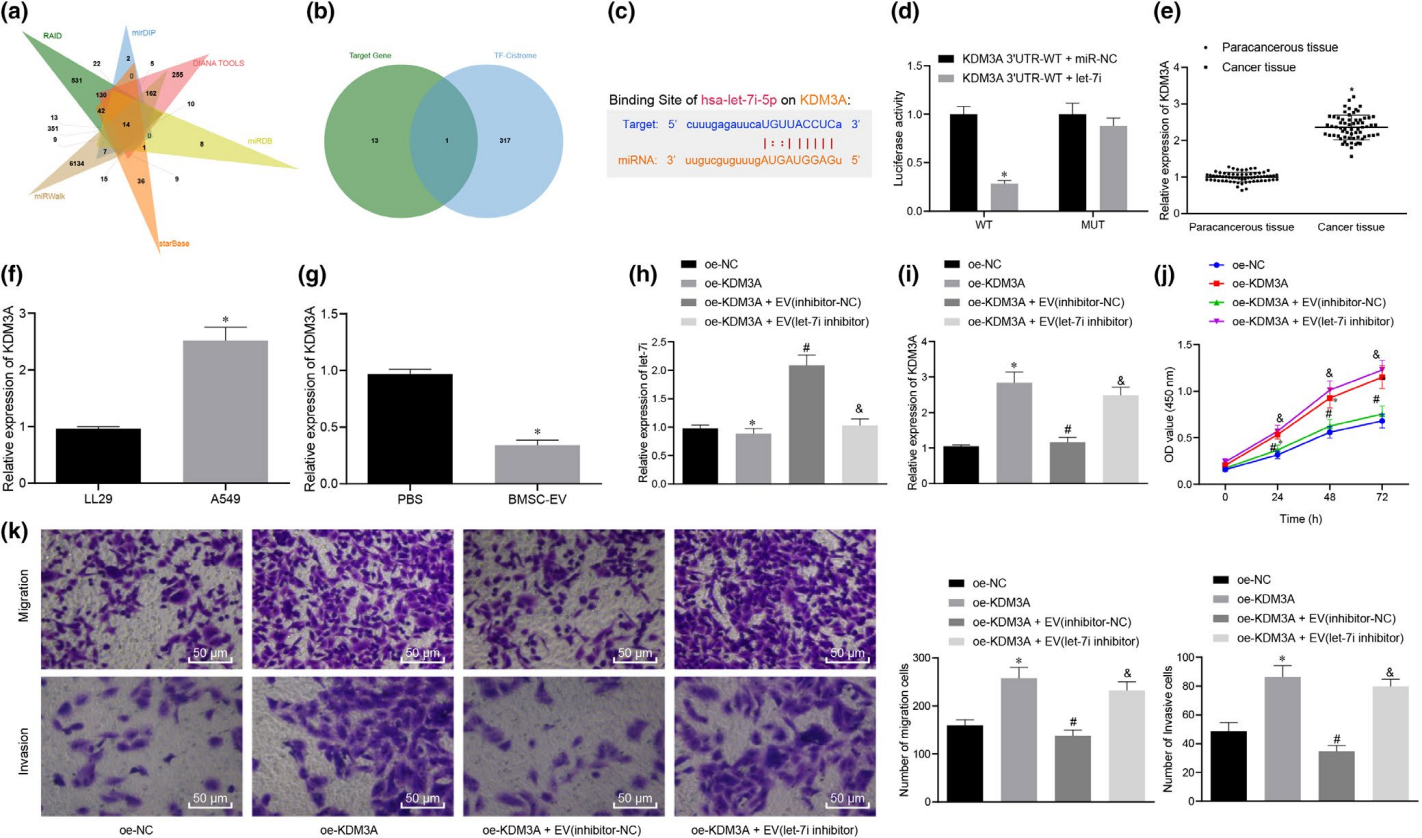
BMSC‐EV‐derived let‐7i represses KDM3A to further inhibit the development of lung cancer. A, RAID, mirDIP, DIANA TOOLS, miRDB, starBase and miRWalk were used to predict the downstream genes of let‐7i and Venn diagrams were plotted to take intersections. B, Venn diagram of human transcription obtained from let‐7i downstream genes and Cistrome Factor and the intersection gene is KDM3A. C, The binding site of let‐7i and KDM3A predicted by starBase. D, The relationship between let‐7i and KDM3A verified by dual‐luciferase reporter gene assay. E, The expression of KDM3A in lung cancer tissues determined by RT‐qPCR (n = 65), *P < .05 compared with paracancerous tissues. F, The expression of KDM3A in lung cancer cells A549 and normal human lung fibroblasts LL29 measured by RT‐qPCR, *P < .05 compared with LL29. G, The expression of KDM3A in lung cancer cells treated with EVs determined by RT‐qPCR, *P < .05 compared with cells treated by BMSC‐EVs. H‐I, The expression of let‐7i and KDM3A in lung cancer cells transfected with oe‐KDM3A treated by EVs measured by RT‐qPCR. *P < .05 compared with cells transfected with oe‐NC, #*P* < .05 compared with cells transfected with oe‐KDM3A; & *P* < .05 compared with cells transfected with KDM3A + EV‐inhibitor‐NC. J, The proliferation ability of lung cancer cells treated with EVs determined by CCK‐8, *P < .05 compared to cells transfected with oe‐NC; #*P* < .05 compared to cells transfected with oe‐KDM3A; & *P* < .05 compared to cells transfected with oe‐KDM3A + EV‐inhibitor‐NC. K, The migration and invasion of lung cancer cells determined by Transwell (× 200), *P < .05 compared with cells transfected with oe‐NC; #*P* < .05 compared with cells transfected with oe‐KDM3A; &*P* < .05 compared with cells transfected with oe‐KDM3A + EV‐inhibitor‐NC. The experiments were repeated 3 times

To further study the downstream regulation mechanism of let‐7i, RAID, mirDIP, DIANA TOOLS, miRDB, starBase and miRWalk were applied and intersections were taken by plotting the Venus diagrams and acquired 14 key genes (Figure 3A), among which the most important transcription factor KDM3A was obtained (Figure 3B). The binding site of let‐7i and KDM3A was predicted by the StarBase (Figure 3C). Results of the dual‐luciferase reporter gene assay demonstrated that the luciferase activity of cells cotransfected with let‐7i and KDM3A WT 3’UTR was significantly decreased, whereas the cells which received other treatment presented with no remarkable changes (Figure 3D). The positive expression rate of the KDM3A in lung cancer tissues and paracancerous tissues was determined by the RT‐qPCR, and KDM3A was found to have a high expression in lung cancer tissues (Figure 3E). Moreover, IHC results further confirmed that KDM3A was overexpressed in lung cancer tissues (Figure S1). The expression of KDM3A in lung cancer cell line A549 was also measured, and the results showed that compared with that in LL29 cells, KDM3A was highly expressed in lung cancer cell line A549 (Figure 3F). Following treatment with EVs, the expression of KDM3A in lung cancer cells was measured. As a result, compared with the treatment of PBS, the expression of KDM3A in lung cancer cells was notably reduced after treated by BMSC‐EVs (Figure 3G).

To further confirm that let‐7i in the BMSC‐EVs is capable of regulating the expression of KDM3A and mediate the proliferation, migration and invasion of lung cancer cells, the lung cancer cells A549 were transfected with oe‐KDM3A. Then, inhibitor‐NC and let‐7i inhibitor were transfected into BMSC and added to lung cancer cells transfected with oe‐KDM3A after extracting BMSC‐EVs. The expression of KDM3A and proliferation, migration and invasion of lung cancer cells was detected. Our results revealed that compared with that of cells transfected with oe‐NC, let‐7i in cells transfected with oe‐KDM3A had no significant changes, whilst the expression of KDM3A was dramatically increased and lung cancer cell proliferation, migration, and invasion ability was promoted. Compared with cells transfected with oe‐KDM3A, let‐7i in cells treated by oe‐KDM3A + EV‐inhibitor‐NC was notably increased; however, the KDM3A expression was significantly down‐regulated, whereas the proliferation, migration and invasion abilities of lung cancer cells were reduced. Moreover, compared with cells treated by oe‐KDM3A + EV‐inhibitor‐NC, the expression of let‐7i in cells treated by oe‐KDM3A + EV‐let‐7i inhibitor was remarkably reduced, KDM3A was evidently up‐regulated, and the proliferation, migration and invasion abilities of lung cancer cells were enhanced (Figure 3H‐K). Collectively, these above‐described results demonstrated that after the addition of EVs to lung cancer cells, KDM3A was down‐regulated, whereas the lung cancer cell proliferation, migration and invasion would be inhibited. On the other hand, when lung cancer cells were added with EV‐let‐7i‐inhibitor, the expression of KDM3A was up‐regulated, thus promoting lung cancer cell proliferation, migration and invasion, suggesting that the BMSC‐EV‐derived let‐7i was a suppressor of KDM3A in lung cancer cells and an inhibitor of the proliferation, migration and invasion of lung cancer cells.

### 3.3 KDM3A promotes DCLK1 expression by removing H3K9me2 modification, thereby promoting proliferation, migration and invasion of lung cancer cells

**Figure 4:**
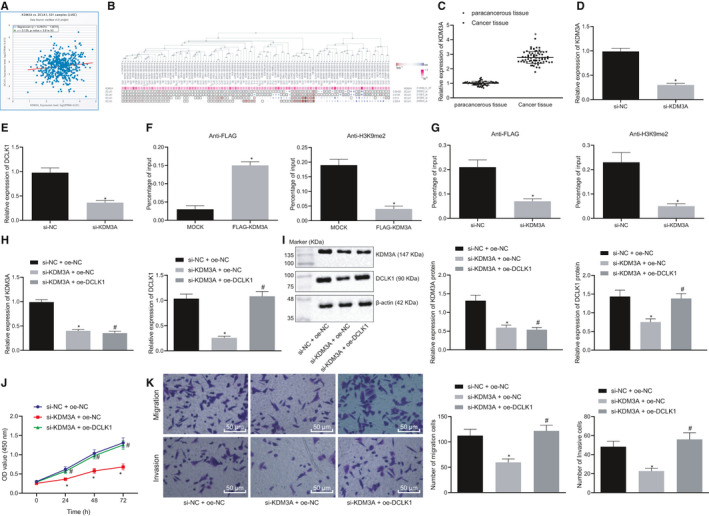
KDM3A promotes DCLK1 expression and regulates proliferation, migration and invasion of lung cancer cells. A, The correlation between KDM3A and DCLK1 expression in lung cancer predicted by StarBase (r = 0.129, *P* = 3.91e‐03). B, The co‐expression relationship between KDM3A and DCLK1 identified by MEM analysis (*P* = 4.23e‐03). C, The expression of KDM3A in lung cancer tissues measured by RT‐qPCR (n = 65), *P < .05 compared with paracancerous tissues. D & E, The expression of KDM3A and DCLK1 in lung cancer cells after the transfection of si‐KDM3A, *P < .05 compared with that of cells transfected with si‐NC. F & G, The changes of histone H3K9 modification in KDM3A and DCLK1 enhancer regions after lung cancer cells transfected with si‐KDM3A determined by ChIP analysis using anti‐H3K9me2 antibody, *P < .05 compared to that of cells transfected with si‐NC. H, The mRNA expression of KDM3A and DCLK1 determined by RT‐qPCR, *P < .05 compared with cells transfected with si‐NC + oe‐NC, #*P* < .05 compared with cells transfected with si‐KDM3A + oe‐NC. I, The protein expression of KDM3A and DCLK1 determined by Western blot analysis, *P < .05 compared with cells transfected with si‐NC + oe‐NC, #*P* < .05 compared with cells transfected with si‐KDM3A + oe‐NC. J, The proliferation of lung cancer cells treated with EVs determined by CCK‐8 assay. *P < .05 compared with cells transfected with si‐NC + oe‐NC, #*P* < .05 compared with cells transfected with si‐KDM3A + oe‐NC. K, The migration and invasion of lung cancer cell determined by Transwell assay (× 200), *P < .05 compared with cells transfected with si‐NC + oe‐NC, #*P* < .05 compared with cells transfected with si‐KDM3A + oe‐NC. The experiment was repeated three times

According to a previous study, both KDM3A and DCLK1 can deteriorate the lung cancer, whilst KDM3A can promote the expression of DCLK1 through its demethylation modification.^21^, ^29^ Therefore, the underlying regulatory mechanism of demethylase KDM3A on DCLK1 was further evaluated in lung cancer. Our results from starBase analysis indicated no significant correlation between the expression of KDM3A and DCLK1 (Figure 4A), whilst MEM analysis illustrated a remarkable co‐expression relationship between KDM3A and DCLK1 (Figure 4B). Then, the expression of DCLK1 in lung cancer tissues and paracancerous tissues was measured with the use of RT‐qPCR, the results of which showed that DCLK1 was highly expressed in the lung cancer tissues (Figure 4C). IHC results further confirmed that DCLK1 was overexpressed in lung cancer tissues (Figure S1).

To determine the regulatory relationship between KDM3A and DCLK1, lung cancer cells were transfected with si‐KDM3A and si‐KDM3A‐1 with high efficiency was selected to detect its effect on the expression of DCLK1 in lung cancer cells. It was found that compared with that of cells transfected with si‐NC, the expression of DCLK1in cells transfected with si‐KDM3A was notably reduced (Figure 4D and E). To explore the binding ability of demethylase KDM3A to the promoter region of DCLK1, lung cancer cells were transfected with 3 × FLAG‐KDM3A vector and ChIP analysis was performed. The results displayed that KDM3A could bind to DCLK1 and histone H3K9 dimethylation in the promoter region of the DCLK1 gene was detected (Figure 4F). To examine the function of endogenous KDM3A protein in lung cancer cells, ChIP analysis was conducted in cells using anti‐H3K9me2 after the treatment of si‐KDM3A. Our data illustrated that si‐KDM3A treatment increased the H3K9me2 in the DCLK1 promoter region (Figure 4G). Therefore, endogenous KDM3A protein can bind to the promoter region of DCLK1 and promote the expression of the DCLK1 gene by removing histone H3K9me2.

To identify that KDM3A regulates lung cancer cell proliferation, migration and invasion through DCLK1, lung cancer cells were transfected with si‐KDM3A + oe‐DCLK1 and the expression of KDM3A and DCLK1 and lung cancer cell proliferation, migration and invasion were determined. The results indicated that compared with the cells transfected with si‐NC + oe‐NC, the expressions of KDM3A and DCLK1 in cells transfected with si‐KDM3A + oe‐NC were reduced, whereas proliferation, migration and invasion of lung cancer cells were notably reduced. Moreover, compared with the cells transfected with si‐KDM3A + oe‐NC, there was no significant change observed in KDM3A expression in cells transfected with si‐KDM3A + oe‐DCLK1, whereas DCLK1 expression was increased and lung cancer cell proliferation, migration and invasion were remarkably increased (Figure 4H‐K). Collectively, these above‐mentioned results supported the fact that KDM3A can promote the expression of DCLK1 by removing the H3K9me2 modification and ultimately enhance the proliferation, migration and invasion of lung cancer cells.

### 3.4 DCLK1 promotes the proliferation, migration and invasion of lung cancer cells by inhibiting FXYD3

**Figure 5:**
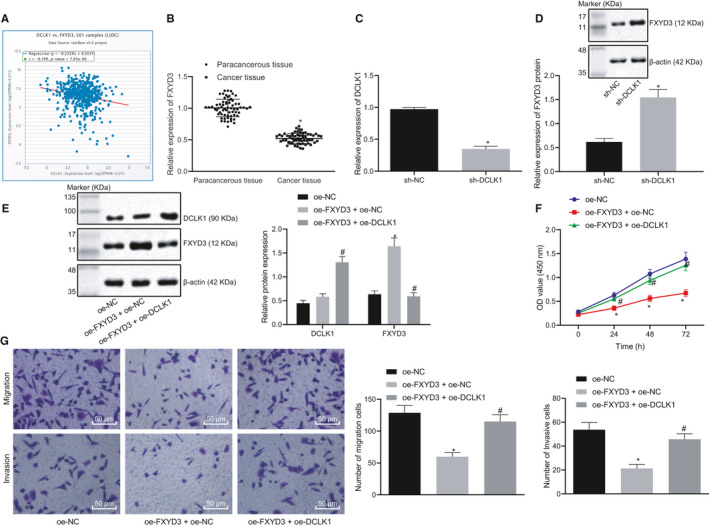
DCLK1 could inhibit FXYD3 and promote the proliferation, migration and invasion of lung cancer cells. A, The correlation between the expression of DCLK1 and FXYD3 in lung cancer predicted in StarBase (r = −0.199, *P* = 7.05e‐06). B, The expression of FXYD3 in lung cancer tissues and paracancerous tissues determined by RT‐qPCR (n = 65), * *P* < .05 compared with paracancerous tissues. C, The mRNA expression of DCLK1 in lung cancer cells after the transfection of sh‐DCLK1 measured by RT‐qPCR, *P < .05 compared with cells transfected with sh‐NC. D, The mRNA expression of FXYD3 in lung cancer cells after transfection of sh‐DCLK1 determined by RT‐qPCR, *P < .05 compared with cells transfected with sh‐NC. E, The protein expression of FXYD3 in lung cancer cells after transfection of oe‐DCLK1 or oe‐FXYD3 determined by Western blot analysis, *P < .05 compared with cells transfected with sh‐NC. F, The proliferation of lung cancer cells treated with EVs determined by CCK‐8, *P < .05 compared with cells transfected with oe‐NC, #*P* < .05 compared with cells transfected with oe‐FXYD3 + oe‐NC. G, The migration and invasion of lung cancer cells detected by Transwell (× 200), *P < .05 compared with cells transfected with oe‐NC, #*P* < .05 compared with cells transfected with oe‐FXYD3 + oe‐NC. The experiment was repeated 3 times

Previously reported research work has confirmed that DCLK1 is an inhibitor of FXYD3.^30^ Thus, we hypothesize that DCLK1 regulates the development of lung cancer by mediating the expression of FXYD3. For this purpose, StarBase was employed to predict the relationship between DCLK1 and FXYD3 and we found that they were negatively correlated (Figure 5A). The expression of FXYD3 in lung cancer tissues and paracancerous tissues was measured with the application of RT‐qPCR, the findings of which showed a low expression of FXYD3 in lung cancer tissues (Figure 5B). IHC results further demonstrated that FXYD3 was overexpressed in lung cancer tissues (Figure S1). Thereafter, the lung cancer cells were transfected with sh‐DCLK1 to detect the knockdown efficiency of DCLK1, whereas sh‐DCLK1 with high transfection efficiency was selected for follow‐up experiments (Figure 5C). It was found that the expression of FXYD3 protein in the cells transfected with sh‐DCLK1 was notably up‐regulated (Figure 5D). To further determine that DCLK1 regulates the lung cancer cell proliferation, migration and invasion through FXYD3, lung cancer cells were transfected with oe‐DCLK1 + oe‐FXYD3 and the results illustrated that the overexpression of FXYD3 led to increased FXYD3 expression but inhibited proliferation, migration and invasion abilities of lung cancer cells; however, these changes could be reversed by the addition of oe‐DCLK1 (Figure 5E‐G). Hence, DCLK1 could inhibit the FXYD3 and promote the proliferation, migration and invasion of lung cancer cells.

### 3.5 BMSC‐EV‐derived let‐7i suppresses the pathogenesis of lung cancer by suppressing DCLK1/FXYD3 axis through KDM3A

**Figure 6:**
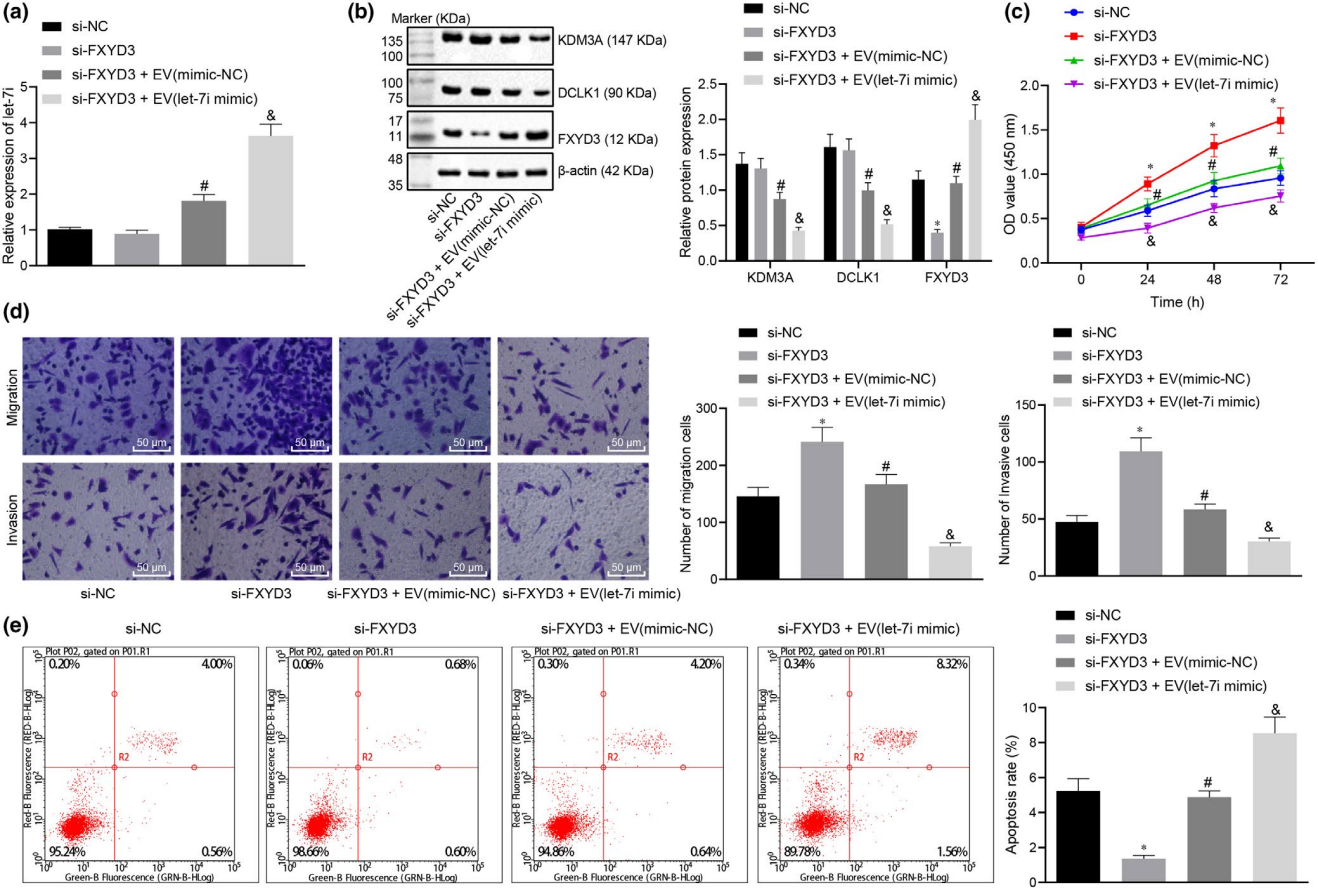
BMSC‐EV‐derived let‐7i can inhibit the DCLK1/FXYD3 axis by regulating KDM3A, thus inhibiting the pathogenesis of lung cancer. A, The expression of let‐7i after co‐culture of si‐NC‐transfected lung cancer cells EVs‐treated lung cancer cells measured by RT‐qPCR, *P < .05 compared with cells transfected with si‐NC, &*P* < .05 compared with cells transfected with si‐FXYD3 + EV‐inhibitor‐NC. B, The KDM3A, DCLK1 and FXYD3 protein expression determined by Western blot analysis, *P < .05 compared with cells transfected with si‐NC, #*P* < .05 compared with cells transfected with si‐FXYD3, &*P* < .05 compared with cells transfected with si‐FXYD3 + EV‐inhibitor‐NC. C, The proliferation of lung cancer cells after EV treatment determined by CCK‐8, *P < .05 compared to cells transfected with si‐NC, #*P* < .05 compared to cells transfected with si‐FXYD3, &*P* < .05 compared to cells transfected with si‐FXYD3 + EV‐inhibitor‐NC. D, The migration and invasion of lung cancer cells after EV treatment determined by Transwell (× 200), *P < .05 compared to cells transfected with si‐NC, #*P* < .05 compared to cells transfected with si‐FXYD3, &*P* < .05 compared with cells transfected with si‐FXYD3 + EV‐inhibitor‐NC. E, Flow cytometry analysis of lung cancer cell apoptosis after EV treatment, *P < .05 compared with cells transfected with si‐NC, #*P* < .05 compared with cells transfected with si‐FXYD, &*P* < .05 compared with cells treated by si‐FXYD3 + EV‐inhibitor‐NC. The experiment was repeated 3 times

To identify that the BMSC‐EV‐derived let‐7i was involved in the occurrence and development of lung cancer by inhibiting the DCLK1/FXYD3 axis through KDM3A, BMSCs were transfected with mimic‐NC or let‐7i mimic and then BMSC‐EVs were extracted and added to lung cancer cells transfected with si‐FXYD3, respectively. Our results showed that compared with cells transfected with si‐NC, the expression of let‐7i, KDM3A and DCLK1 in cells transfected with si‐FXYD3 manifested no significant changes; however, the expression of FXYD3 was notably reduced. Moreover, the proliferation and invasion of lung cancer cells was increased and the number of apoptosis was reduced. Compared with cells transfected with si‐FXYD3, the expression of let‐7i and FXYD3 was enhanced in cells treated with si‐FXYD3 + EV‐mimic‐NC, the expression of KDM3A and DCLK1 was decreased, whereas the proliferation and invasion of lung cancer cells were inhibited, and the apoptosis was promoted. Compared with cells treated by si‐FXYD3 + EV‐mimic‐NC, let‐7i and FXYD3 expression in cells treated by si‐FXYD3 + EV‐let‐7i mimic were significantly increased, the expressions of KDM3A and DCLK1 were noticeably down‐regulated, whereas the proliferation and invasion of lung cancer cells were inhibited remarkably, whereas the number of apoptotic cells was significantly increased (Figure 6A‐E). Hence, BMSC‐EV‐derived let‐7i was identified as an inhibitor of the pathogenesis of lung cancer by suppressing the DCLK1/FXYD3 axis through KDM3A.

### 3.6 BMSC‐EV‐derived let‐7i inhibits the pathogenesis of lung cancer cells in vivo through KDM3A/DCLK1/FXYD3 axis

**Figure 7:**
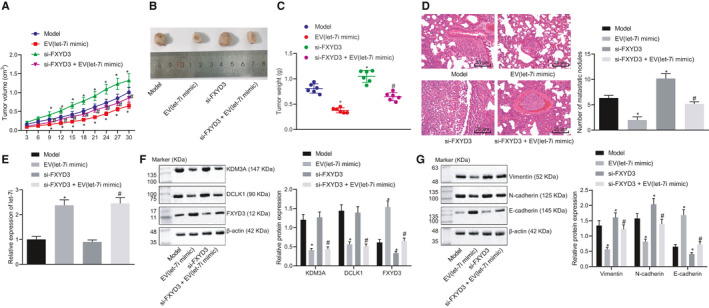
Let‐7i in BMSC suppresses proliferation and metastasis of lung cancer cells in nude mice. A, Statistical analysis of tumour volume of nude mice at various time‐points (n = 6). B & C, Representative pictures of tumours and statistical analysis of tumour weight of nude mice on the 30th d. D, Statistical analysis of nodule metastasis in lung tissues detected by HE staining (× 400). E & F, The expression of let‐7i, KDM3A, DCLK1, and FXYD3 in tumour tissues determined by RT‐qPCR and Western blot analysis. G, The expression of vimentin, N‐cadherin and E‐cadherin in tumour tissues determined by Western blot analysis. *P < .05 compared to model mice, #*P* < .05 compared to nude mice treated by si‐FXYD3. Data are all measurement data and expressed as the mean ± standard deviation

To study the effect of let‐7i on the growth of lung cancer cells in vivo through the KDM3A/DCLK1/FXYD3 axis, A549 cells that received differing treatment were inoculated into nude mice subcutaneously to establish a xenograft model. After 30 days, the size and weight of the tumour in nude mice were measured. Intriguingly, our results illustrated that compared with model mice, tumours in nude mice treated by EV‐let‐7i‐mimic were notably reduced (*p* ˂ 0.05), whereas tumours in nude mice treated by si‐FXYD3 were considerably increased (*p* ˂ 0.05). Although compared with si‐FXYD3 treatment, tumours in nude mice treated by si‐FXYD3 + EV‐let‐7i mimic were sharply decreased (*p* ˂ 0.05) (Figure 7A‐C). Moreover, the lung tissue of nude mice was observed by the HE staining and the number of metastatic tumour nodules formed in the lung was counted. Our results denoted that compared with model mice, the lung tissue damage in mice treated by EV‐let‐7i mimic was reduced with a relatively decreased number of metastatic tumour nodules (*p* ˂ 0.05), whereas the lung tissue of nude mice transfected with si‐FXYD3 was severely damaged with a relatively increased number of metastatic tumour nodules (*p* ˂ 0.05). Compared with mice injected with cells expressing si‐FXYD3, decreased lung tissue damage and the number of metastatic tumour nodules was found after the si‐FXYD3 + EV‐let‐7i‐mimic treatment (Figure 7D).

RT‐qPCR and Western blot analyses were conducted to determine the expression of let‐7i, KDM3A, DCLK1 and FXYD3 in tumours. Our results illustrated that compared with model mice, let‐7i and FXYD3 expression in mice treated by EV‐let‐7i mimic was increased; however, the KDM3A and DCLK1 expressions were inhibited (*p *˂ 0.05). Besides, let‐7i, KDM3A and DCLK1 expressions in mice treated by si‐FXYD3 exhibited no significant change but the expression of FXYD3 was dramatically reduced (*p *˂ 0.05). Moreover, compared with si‐FXYD3 treatment, let‐7i and FXYD3 expression in mice treated by si‐FXYD3 + EV‐let‐7i‐mimic was increased, whereas KDM3A and DCLK1 expression was inhibited and the expression of FXYD3 was notably up‐regulated (*p *˂ 0.05) (Figure 7E and F). Subsequently, the expression of mesenchymal markers, that is vimentin, N‐cadherin and epithelial marker E‐cadherin in tumour tissues, was also measured by Western blot analysis. Our results revealed that compared with model mice, the expression of vimentin and N‐cadherin in mice treated by EV‐let‐7i mimic was notably decreased, whereas the expression of E‐cadherin was significantly increased (*p* ˂ 0.05), whilst the expression of vimentin, N‐cadherin was elevated, and the expression of E‐cadherin was dramatically dropped after si‐FXYD3 treatment (*p* ˂ 0.05). Moreover, compared with mice treated by si‐FXYD3, the expression of vimentin in mice treated by si‐FXYD3 + EV‐let‐7i mimic was markedly reduced, whereas E‐cadherin expression was remarkably increased (*P* < .05) (Figure 7G). Therefore, let‐7i in BSMC‐EVs exerted a repressing effect on the progression of lung cancer in vivo.

## 4 DISCUSSION

**Figure 8:**
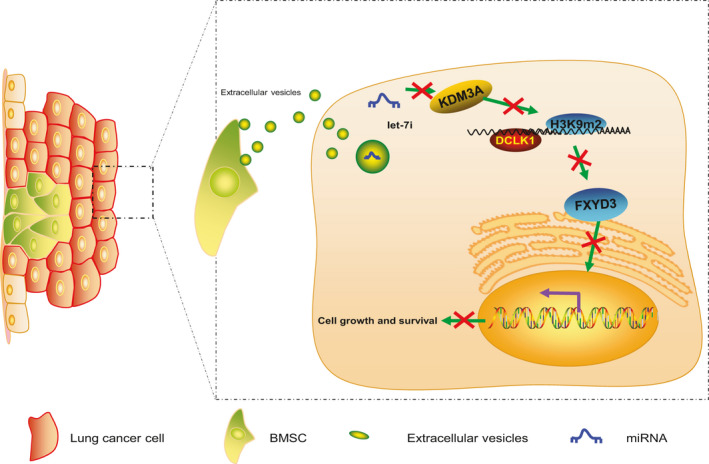
BMSC‐EV‐derived let‐7i repressed the proliferation, migration and invasion of lung cancer cells by decreasing the FXYD3 *via* elevating DCLK1 through KDM3A inhibition, thus representing an effective suppressor of lung cancer development

BMSCs have been proposed as crucial regulators of endogenous processes such as hematopoiesis, and their involvement in the survival of tumours has been previously highlighted, implicating them as promising therapeutic targets for oncologic diseases.^31^ Moreover, EVs are released from all kinds of cell types and modulate cell‐cell communication, and have emerged as biomarkers and therapeutic targets for lung cancer.^32^ Peculiarly, BMSC’s secretome is designated as the suppressor of lung cancer progression and an enhancer of a patient's survival.^33^ Accordingly, the current study was designed to investigate the particular function and molecular mechanism underlying the role of BMSC‐EV‐derived let‐7i on lung cancer and our results illustrated that the BMSC‐EV‐derived let‐7i led to the suppression of proliferation, migration and invasion of lung cancer cells by decreasing the FXYD3 by elevating DCLK1 via KDM3A inhibition, thus potentially serving as an effective suppressor of lung cancer development (Figure 8).

Primarily, our study revealed that lung tissues and cells have a poor expression of let‐7i, whereas it was highly expressed in BMSC‐EVs. A previous study has reported that multiple non‐coding RNAs are altered by polyamine depletion including induction of microRNA let‐7i, a member of the tumor suppressive let‐7 family.^34^ Consistent with our findings, it has been reported that lung cancer tissues and cells also have low expression of let‐7.^35^, ^36^ Furthermore, the low expression of let‐7 in lung cancer has been linked with the poor prognosis of lung cancer patients.^37^ Moreover, let‐7 has been found to be highly expressed miRNA in EVs derived from human adipose tissue stem cells.^38^, ^39^ Lung cancers were then treated with BMSC‐EV‐let‐7i‐mimic to further explore the effect of let‐7i in BMSC‐EVs on lung cancer, the results of which showed significant suppression of proliferation, migration and invasion of lung cancer cells. Similarly, EVs released by BMSCs have indicated to be effective in alleviating inflammation and injury.^40^ Moreover, EVs produced by MSCs have been reported to serve vital therapeutic roles in the clinical treatment of inflammatory lung diseases.^28^ Importantly, EVs targeted let‐7 miRNA can be delivered to breast cancer cells and inhibit tumour growth.^41^ Therefore, we predicted that let‐7i in BMSC‐EVs could be a promising inhibitor of lung cancer.

Subsequently, our results uncovered an aberrantly high expression of KDM3A in lung cancer cells whereas the repression of KDM3A induced by let‐7i resulted in the inhibition of lung cancer development. Moreover, KDM3A, a histone demethylase in the JmjC domain‐containing protein family, is known to be up‐regulated in tumours and exerts a pro‐tumorigenic function.^19^, ^20^, ^42^ For instance, KDM3A expression was notably increased in colorectal cancer metastatic lesions and its high expression was correlated with poor prognosis and short overall survival of colorectal cancer patients.^43^ Accordingly, previous studies have showed the presence of a high expression of KDM3A in lung cancer cells, and thus, it is considered to be responsible for the development of lung cancer.^22^

Furthermore, our results illustrated that KDM3A promotes the DCLK1 expression by binding to DCLK1 promoter and removing H3K9me2 modification, thereby promoting proliferation, migration and invasion of lung cancer cells. DCLK1 has also been proposed as a novel cancer stem cell marker in a plethora of human cancers.^44^ According to previous studies, the overall survival of lung cancer patients was remarkably reduced after methylation of DCLK1 promoter, which was consistent with our findings.^45^ Particularly, KDM3A has been reported to enhance the expression of DCLK1 with the removal of di‐ and mono‐methyl residues from H3K9me2/me1, and their high expression in pancreatic cancer tissues was correlated with shorter survival times of patients.^21^ Lastly, we found that the DCLK1 promotes the proliferation, migration and invasion of lung cancer cells through the inhibition of FXYD3. Accordingly, FXYD3, being a sodium‐potassium ATPase regulator, has been confirmed as a key mediator in a large number of cancer types.^26^ Meanwhile, the poor expression of FXYD3 has been previously reported in lung cancer cells whereas its inactivation was identified as a potential player in lung cancer development.^27^ Another study has demonstrated that the expression of FXYD3 was reduced in colorectal cancer cells with overexpression of DCLK1, thus representing a novel therapeutic target for colorectal cancer.^30^ Collectively, these findings illustrated KDM3A as a tumour promoter in lung cancer through FXYD3 suppression by elevating DCLK1 via the removal of the methylation of H3K9me2 in the DCLK1 promoter region.

In summary, our study provided evidence in support of the suppressive effects of BMSC‐EV‐derived let‐7i on the development of lung cancer cells through FXYD3 down‐regulation by increasing DCLK1 via KDM3A inhibition. However, the underlying mechanism of let‐7i in lung cancer requires additional investigations. Taken together, the aforementioned findings validated the antitumour effect of BMSC‐EV‐derived let‐7i, which could be promising in its use as a clinically viable target in lung cancer treatment.

## CONFLICTS OF INTEREST

The authors declare no conflict of interest.

## AUTHOR CONTRIBUTION

**Jiefeng Liu:** Conceptualization (equal); Methodology (equal). **Yuhua Feng:** Formal analysis (equal); Supervision (equal). **Xinyu Zeng:** Resources (equal); Validation (equal). **Miao He:** Investigation (equal); Software (equal). **Yujing Gong:** Investigation (equal); Writing‐original draft (equal). **Yiping Liu:** Data curation (equal); Writing‐review & editing (equal).

## Supporting information

Fig S1

## Data Availability

The data that support the findings of this study are available from the corresponding author upon reasonable request.
